# Yes-Associated Protein (YAP) Modulates Oncogenic Features and Radiation Sensitivity in Endometrial Cancer

**DOI:** 10.1371/journal.pone.0100974

**Published:** 2014-06-27

**Authors:** Masahiro Tsujiura, Virginia Mazack, Marius Sudol, Hanna G. Kaspar, John Nash, David J. Carey, Radhika Gogoi

**Affiliations:** 1 Weis Center for Research, Geisinger Medical Center, Danville, Pennsylvania, United States of America; 2 Icahn School of Medicine at Mount Sinai, New York, New York, United States of America; 3 Geisinger Medical Laboratories, Geisinger Wyoming Valley, Wilkes-Barre, Pennsylvania, United States of America; 4 Division of Obstetrics and Gynecology, Geisinger Medical Center, Danville, Pennsylvania, United States of America; Institute of Molecular and Cell Biology, Biopolis, United States of America

## Abstract

**Background:**

Yes-associated protein (YAP) is a transcriptional co-activator and regulates cell proliferation and apoptosis. We investigated the clinical and biological significance of YAP in endometrial cancer (EMCA).

**Methods:**

YAP expression in 150 primary tumor tissues from patients with EMCA was evaluated by immunohistochemistry and its association with clinicopathological data was assessed. The biological functions of YAP were determined in EMCA cell lines through knockdown/overexpression of YAP. The role of YAP in modulating radiation sensitivity was also investigated in EMCA cells.

**Results:**

Increased nuclear YAP expression was significantly associated with higher grade, stage, lympho-vascular space invasion, postoperative recurrence/metastasis and overall survival in estrogen mediated EMCA, called type 1 cancer (p = 0.019,  = 0.028,  = 0.0008,  = 0.046 and  = 0.015, respectively). In multivariate analysis, nuclear YAP expression was confirmed as an independent prognostic factor for overall survival in type 1 EMCA. YAP knockdown by siRNA resulted in a significant decrease in cell proliferation (p<0.05), anchorage-dependent growth (p = 0.015) and migration/invasion (p<0.05), and a significant increase in the number of cells in G0/G1 phase (p = 0.002). Conversely, YAP overexpression promoted cell proliferation. Clonogenic assay demonstrated enhanced radiosensitivity by approximately 36% in YAP inhibited cells.

**Conclusions:**

Since YAP functions as a transcriptional co-activator, its differential localization in the nucleus of cancer cells and subsequent impact on cell proliferation could have important consequences with respect to its role as an oncogene in EMCA. Nuclear YAP expression could be useful as a prognostic indicator or therapeutic target and predict radiation sensitivity in patients with EMCA.

## Introduction

Endometrial cancer (EMCA) is the fourth most common cancer and the most common gynecologic cancer in American women, with approximately 8200 deaths and 49500 new cases in the United States in 2013 [1]. While women with EMCA generally have a good prognosis with 81.5% 5-year survival (2003–2009), the incidence and death rate of EMCA have continued to rise on average 1.1% and 0.4% respectively each year over the last 10 years [2]. Recent increases in the incidence of endometrial cancer rates have been considered largely attributed to the obesity epidemic [3]. Although improvements in diagnostic techniques and peri-operative management have resulted in an increase in the early detection of EMCA and favorable prognosis, women diagnosed with advanced or recurrent disease have much worse survival rates and limited adjuvant treatment options. Gene expression studies have identified some genes that are differentially expressed in EMCA, such as *PTEN*, *KRAS*, *CTNNB1*, *PIK3CA* and *FGFR2* [4], [5], [6], [7]. In the clinical setting, however, few molecules have been assayed as therapeutic and/or diagnostic biomarkers. Therefore, identification of biologic markers and their downstream targets is essential for personalizing therapies and improving the outcome and quality of life in patients with EMCA.

EMCA has been categorized into two types. Type 1 cancer accounts for approximately 80% of EMCA and is characterized as estrogen dependent, estrogen receptor (ER) and progesterone receptor (PR) positive with endometrioid morphology and generally a favorable prognosis [8]. Conversely, type 2 cancer is estrogen-independent, ER/PR negative, poorly differentiated and associated with a much poorer prognosis [9]. Standard therapy for both types includes a surgical removal of the uterus, cervix, bilateral fallopian tubes and ovaries. Patients whose tumors demonstrate high risk features may additionally undergo lymphadenectomy. Early-stage EMCA with high risk features, such as deep myometrial invasion, lympho-vascular space invasion (LVSI) and high grade is associated with a 15–25% risk of recurrence [10]. Adjuvant radiotherapy, the most common form of therapy for early-stage high risk patients, has been found in multiple studies to decrease pelvic and vaginal recurrence from 12–14% without therapy to 3-4% with therapy, but still without a corresponding increase in overall survival [11], [12]. In other words, radiotherapy exposes a large number of women to toxicity without any clear benefit in overall mortality, especially the majority of patients who will be free of disease in the absence of additional therapy. Radiation therapy is also used for previously untreated patients with local/regional recurrence. However, despite radiation, 50% of patients with local recurrence will ultimately die of their disease [13], implying that these patients have radiation resistant tumors and may have benefitted from alternate treatment such as chemotherapy. Identifying markers of radiation sensitivity/resistance would allow for tailored therapy to the most effective regimen and decrease unnecessary radiation-induced toxicity.

Yes-associated protein (YAP) was first identified by virtue of its ability to associate with Yes and Src protein-tyrosine kinases [14]. The *YAP* gene is located at human chromosome 11q22, encodes a transcriptional co-activator and is one of the two main downstream effectors of the Hippo tumor suppressive pathway [15]. Inhibition of the Hippo pathway leads to YAP activation, nuclear localization and increased activity of transcriptional target genes, such as *CTGF* and *AREG* [16], [17]. Conversely, activation of the Hippo pathway leads to YAP phosphorylation, cytoplasmic sequestration and inactivation. The YAP serine 127 to alanine (S127A) mutant is a constitutively active form that remains in the nucleus and is transcriptionally active. YAP regulates the balance between cell proliferation and apoptosis [18], [19], [20], and is amplified in a number of human malignancies including breast, esophageal, hepatocellular, ependymoma, malignant mesothelioma and medulloblastoma [21], [22], [23], [24], [25], [26]. In addition, YAP expression correlates with poor prognosis in various cancers, such as colorectal, esophageal, gastric, hepatocellular, lung and ovarian [22], [27], [28], [29], [30], [31], [32],

There is also crosstalk between YAP and steroid hormones. Dhananjayan et al. demonstrated that YAP and the WW domain binding protein-2 (WBP-2) are co-activators of ER and PR [33], which play a key role in the normal menstrual cycle and in the etiology of type 1 EMCA. Although several reports demonstrated oncogenic functions for YAP in various human cancers, its biological and clinical relevance in EMCA remains unclear. In addition, YAP overexpression promotes radioresistance in medulloblastoma cells through the YAP/IGF2/Akt pathway [34], suggesting YAP can function in modulating radiation sensitivity/resistance. Those findings urged us to further investigate whether YAP could play a role in oncogenesis and development of EMCA and modulation of radiation sensitivity.

In the present study, we investigated the potential utility of YAP as a prognostic and therapeutic indicator in EMCA and the biological function of YAP in EMCA. Furthermore, we evaluated YAP effect regarding radiation sensitivity. Consequently, our data provide evidence that nuclear YAP expression is a marker for poor prognosis and may have therapeutic implications for the treatment of patients with EMCA.

## Materials and Methods

### Patients and tissue samples

The study was approved by the institutional review board at Geisinger Medical Center (Geisinger Medical Center IRB Protocol #2011–0163) and a waiver of consent was obtained from the IRB Center to perform the study. A total of 150 primary EMCA tissues were obtained from patients who underwent a hysterectomy at Geisinger Medical Center between 2008 and 2011, including 120 cases of type 1 EMCA and 30 cases of type 2 EMCA. Demographic and prognostic information, including age, body mass index (BMI), grade, stage, LVSI and survival data was obtained from all subjects. Macroscopic and microscopic classifications of tumors were based on the International Federation of Gynecologist and Obstetricians (FIGO) staging system [35], [36].

### Immunohistochemistry

Paraffin embedded tissue specimens were cut to a thickness of 5 microns and subjected to immunohistochemical staining of YAP protein with the avidin-biotin-peroxidase method. Antigen retrieval was performed by heating the samples in citrate buffer (pH 6.0) for 5 minutes on high power, then 10 minutes medium power, using a microwave oven. Slides were cooled and rinsed in distilled water, and stained using the DAKO Autostainer as follows: Slides were incubated in 3% hydrogen peroxide 5 minutes with subsequent rinsing in TBST buffer. Samples were then incubated in YAP antibody (1∶200, NB110-58358) from Novus Biologicals (Littleton, CO) for 30 min with a subsequent TBST wash and incubated in Dako Envision+ HRP rabbit solution (Dako, Carpinteria, CA) (diluted according to manufacturer's instructions) for 30 minutes. After a TBST wash, slides were then incubated in Dako DAB solution for 10 minutes and rinsed again with TBST buffer. Slides were then counterstained as follows: 20 second incubation in Gills Hematoxylin stain, followed by a tap water wash. Slides were dehydrated and then air-dried, and specimens were mounted in mounting medium. Immunostained slides were evaluated for both nuclear and cytoplasmic glandular staining of YAP by a gynecological pathologist, (H.K.), scoring for intensity using a 5 point (0 to 4) scale.

### EMCA cell lines

Three human type 1 EMCA cell lines, HEC-1-A, HEC-1-B and Ishikawa, were used in this study. HEC-1-A (ATCC HTB112) and HEC-1-B (ATCC HTB113) were obtained from the American Type Culture Collection (ATCC) (Manassas, VA). Ishikawa cells were provided by Dr. Jennifer Richer (University of Colorado) [37], [38], [39]. HEC-1-A was cultured in McCoy's 5A medium (ATCC, Manassas, VA) supplemented with 10% (v/v) fetal bovine serum (FBS) (Thermo Fisher Scientific, Waltham, MA), HEC-1-B in Eagle's minimum essential medium (EMEM) (ATCC, Manassas, VA) supplemented with 10%(v/v) FBS and Ishikawa in Minimal essential medium (MEM) (Life Technologies, Carlsbad, CA) supplemented with 1% non-essential amino acids (NEAA), 6 ng/ml insulin and 5% (v/v) FBS. Antibiotics (10 units/ml of penicillin and 10 µg/ml of streptomycin) were added to all culture media. All cell lines were incubated at 37°C in a humidified atmosphere containing 5% carbon dioxide.

### Western blot analysis

Cells were lysed in Tris buffer (10 mmol/l, pH 7.4) containing 5 mmol/l EDTA, 300 mmol/l NaCl, 10% glycerol, 1% Triton X-100, 1% sodium deoxycholate, 0.1% SDS and a protease inhibitor cocktail (Roche Diagnostics, Mannheim, Germany) and subjected to SDS-PAGE. Anti-YAP antibody (NB110-58358) was purchased from Novus Biologicals (Littleton, CO); anti-phospho-YAP (ser127) (#4911) from Cell Signaling Technology (Danvers, MA); anti-NF2 (sc-331) from Santa Cruz; anti-LATS2 (ab70565) and anti-GAPDH (ab9485) from Abcam (Cambridge, United Kingdom). Appropriate horseradish peroxidase-conjugated secondary antibodies (Anti-Rabbit/Mouse, GE Healthcare, Little Chalfont, UK) were used. Protein bands were visualized with an enhanced chemiluminescence substrate (Pierce Biotechnology, Rockford, IL) and detected using LAS-3000 (Fujifilm, Tokyo, Japan).

### Immunofluorescence and imaging

Cultured cells were washed with PBS and fixed in 4% (w/v) paraformaldehyde. After permeabilization with 0.2% Triton X-100 in PBS, cells were blocked in 5% (w/v) goat serum in PBS and incubated with primary antibody (YAP, 1∶500) at room temperature for 1 hour, followed by Alexa Fluor 488 goat anti rabbit (1∶500) as secondary antibody for 30 min at room temperature. After being mounted with 4′,6-diamidino-2-phenylindole (DAPI) for nucleus staining, cells were examined using a fluorescence microscope (Olympus IX81, Olympus, Tokyo, Japan).

### Short interfering RNA (siRNA) treatment

The siRNA targeting the *YAP* gene (sc-38637; Santa Cruz Biotechnology, Dallas, TX) was used for loss-of-function experiments. The control siRNA (sc-37007; Santa Cruz Biotechnology, Dallas, TX) was used as a negative control. Each siRNA (37.5 nM) was transfected into EMCA cells using Lipofectamine RNAiMAX (Invitrogen, Carlsbad, CA) according to the manufacturer's instructions. The knockdown of a target gene was verified by western blotting.

### Cell proliferation assay

The numbers of viable cells at various time points after transfection of siRNAs were assessed by a colorimetric water-soluble tetrazolium salt assay (Cell counting kit-8; Dojindo, Kumamoto, Japan) as described elsewhere [40].

### Soft agar assay

For in vitro testing of anchorage-independent colony development, 5000 cells transfected with siRNAs for 48 hours were plated in 1 ml of 0.4% melted agar in EMEM with 10%(v/v) FBS in 6-well plates overlayed with 1.5 ml of 0.9% melted agar in the same medium. Plates were incubated at 37°C in a humidified atmosphere containing 5% carbon dioxide. Plating was performed in sextuplicate and a small amount of EMEM complete medium was carefully added every few days on the top of each well to ensure nutritive supplies and to prevent drying. After incubation for approximately four weeks, cells were fixed and stained with a solution containing 2% ethanol and 0.03% crystal violet and the number of colonies was evaluated by two blinded independent investigators.

### Invasion and Migration Assays

Transwell migration and invasion assays were carried out using 24-well BioCoat cell culture inserts (BD, Franklin Lakes, NJ). The upper surface of 6.4-mm diameter filters with 8-µm pores pores were precoated with (invasion assay) or without (migration assay) extracellular matrix coating (Matrigel). 50,000 siRNA transfected cells in serum-free medium were seeded on to the upper chamber of each insert, with complete medium added to the bottom chamber. Following 24 h of incubation, migrated or invasive cells on the lower surface of the filters were fixed and stained with the Differential Quik Stain Kit (Electron Microscopy Sciences, Hatfield, PA), and numbers of stained cells that had migrated/invaded to the bottom of the filter were counted directly by two independent blinded investigators.

### Cell cycle analysis

For flow cytometric analysis, cells were trypsinized 72 or 96 hours after transfection of siRNAs, followed by incubation in a staining buffer (0.1% of Triton X-100, 0.2 mg/ml RNase A, and 40 µg/ml propidium iodide in PBS). Cells were analyzed for DNA content using Beckman Coulter, Cytomics FC 500 (Brea, CA).

### Expression constructs and colony formation assay

Plasmids expressing wild-type YAP (pEGFP-C3-WT-YAP) were obtained by cloning the full coding sequence of YAP with 504 amino acids [41] into the vector pEGFP-C3 (a gift from Gregory Matera and Channing Der, University of North Carolina, Chapel Hill, NC). S127A mutant form of YAP (pEGFP-C3-S127A-YAP) was generated through PCR-mediated site-directed mutagenesis of pEGFP-C3-WT-YAP. pEGFP-C3-WT-YAP, pEGFP-C3-S127A-YAP or the empty vector (pEGFP-C3-EV) as a control was introduced into EMCA cells using Lipofectamine LTX and Plus Reagent (Invitrogen, Carlsbad, CA) according to the manufacturer's instructions. The expression of YAP protein in transfected cells was confirmed by western blotting. After incubation for approximately four weeks with appropriate concentrations of G418, cells were fixed and stained with a solution containing 10% formaldehyde and 1% crystal violet. The stained area was calculated by densitometry using ImageJ software.

### Clonogenic assay and irradiation conditions

Survival following radiation exposure was defined as the ability of the cells to maintain their clonogenic capacity. Briefly, increasing numbers of cells transfected with the control siRNA or YAP-specific siRNA for 48 hours were plated in 6-well plates. Cells were irradiated with 0 to 6 Gy using a linear accelerator and returned to the incubator for several weeks until cells in control wells had formed sufficiently large colonies. Colonies formed were fixed and stained with a solution containing 10% formaldehyde and 1% crystal violet and those with at least 50 cells were counted by two independent blinded investigators. The number of colonies obtained from three replicates was averaged for each condition. These mean values were corrected according to plating efficiency of respective controls to calculate cell survival for each dose level. The linear quadratic equation was fitted to data sets to generate survival curves, and dose enhancement factor was calculated at 10% surviving fraction (DEF 0.1).

### Statistical analysis

Correlations between nuclear/cytoplasmic staining levels and clinicopathological factors were assessed by the Student's t-test, the Chi square test or the Fisher's test accordingly. The Mann-Whitney *U*-test and the Student's t- test were used to compare the difference in biological behavior between transfected cells and control cells. For the analysis of survival, the Kaplan-Meier survival curves were constructed for groups based on univariate predictors and differences between the groups were tested with the log-rank test. For those variables being statistically significant in the univariate analysis, the Cox proportional hazards model with the likelihood ratio test was used for further evaluation of multivariate survival analysis. P-value <0.05 was considered significant. Statistical analyses were conducted using JMP 10 (SAS Institute Inc., Cary, NC).

## Results

### YAP protein expression in primary EMCA tissues

**Figure 1:**
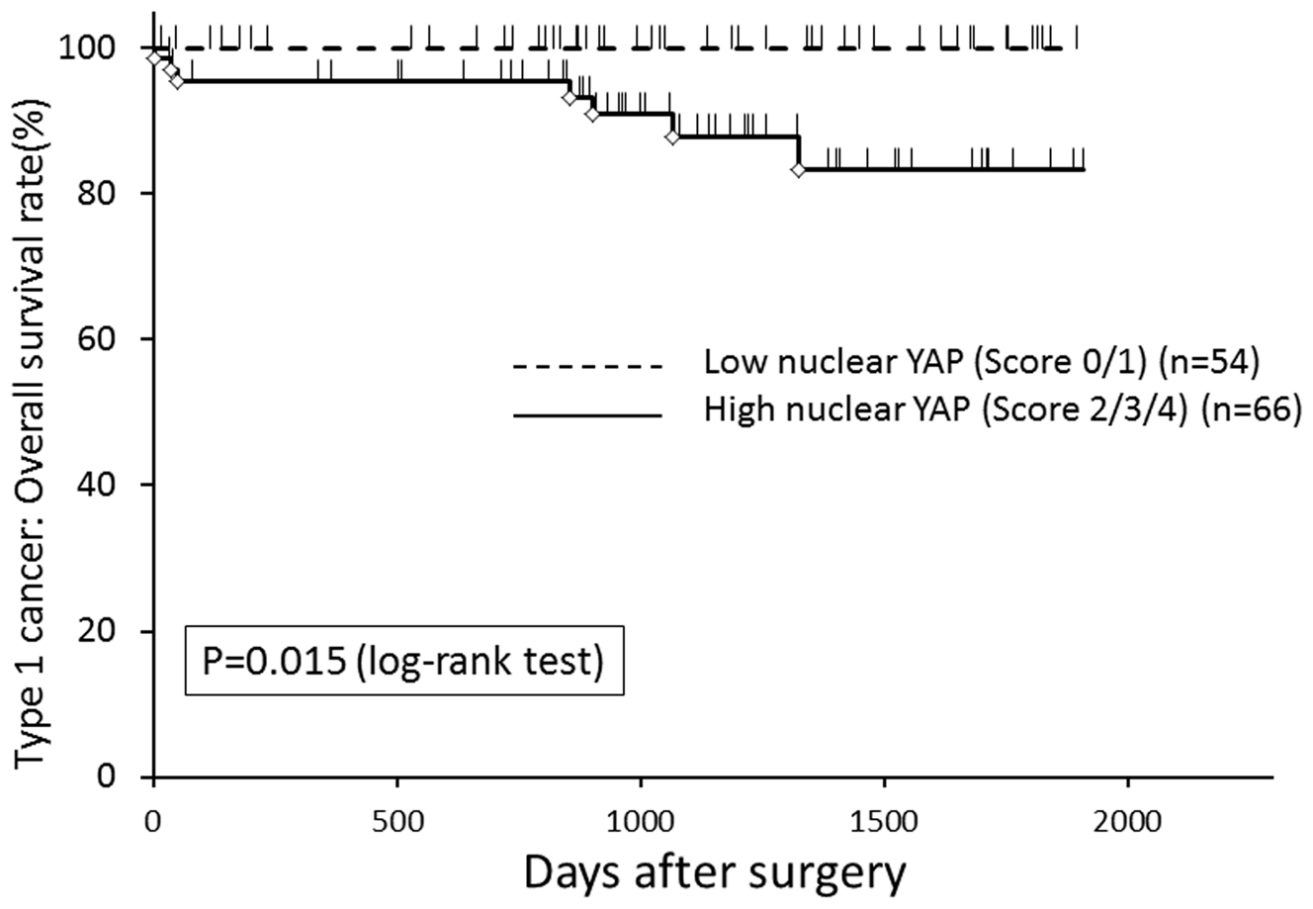
The Kaplan-Meier curve for overall survival rate of patients with type 1 EMCA (n = 120) according to the nuclear expression of YAP. Increased nuclear immunoreactivity of YAP was significantly associated with worse overall survival (P = 0.015, log-rank test).

To determine whether YAP is expressed in human EMCA, YAP protein expression in primary EMCA tissues was assessed by immunohistochemistry. Representative micrographs demonstrating nuclear and cytoplasmic YAP expression are shown in Figure S1. Since the intracellular localization of YAP is generally considered as important for its oncogenic function [42], both the nuclear and cytoplasmic staining levels were analyzed and scored for intensity using a 5 point scale (0 to 4). Demographic data and YAP staining levels comparing type 1 and 2 EMCA are summarized in Table 1. Type 2 EMCA had higher stage, higher frequency of LVSI and postoperative metastasis/recurrence, and higher nuclear YAP expression compared with type 1 EMCA. We next evaluated the correlation between clinicopathological factors and YAP staining in type 1 and type 2 EMCA, individually. As shown in Table 2, higher nuclear YAP expression was significantly associated with higher grade, pathological stage, LVSI and postoperative recurrence/metastasis in type 1 EMCA (p = 0.019,  = 0.028,  = 0.0008,  = 0.046, respectively). No significant association between nuclear YAP expression and these variables was observed in type 2 EMCA (Table S1). Cytoplasmic expression of YAP was not associated with clinicopathological factors in either type 1 or type 2 cancers (**data not shown**). Kaplan-Meier survival estimates showed that increased nuclear immunoreactivity of YAP was significantly associated with worse overall survival in type 1 cancer. (p = 0.015, log-rank test, Figure 1). Univariate analysis indicated advanced stage and presence of LVSI also correlated with poor overall survival (p = 0.0005 and 0.003, log-rank test, respectively, Figure S2). There was no correlation between overall survival and YAP cytoplasmic staining in type 1 EMCA or YAP nuclear/cytoplasmic stain in Type 2 EMCA (Figure S3). By multivariate Cox proportional hazards regression analysis that considered stage, LVSI and nuclear YAP level, only nuclear YAP level (score 2/3/4 versus score 0/1) was independently associated with overall survival (p<0.021). Our data demonstrated that nuclear YAP is associated with poor prognostic features and decreased overall survival in Type 1 EMCA.

**Table 1:** Comparison of demographic data and YAP expression between type 1 and type 2 EMCA.

		Type1 cancer (n = 120)	Type2 cancer (n = 30)	P-value
Age (year)	mean (range)	63.6 (40–89)	67.4 (47–85)	N.S.(a)
BMI	mean (range)	36.8 (19.1–70.4)	35 (18.4–85.4)	N.S.(a)
Ethnic group	White	119	29	
	Other races	1	1	N.S.(b)
Grade	1	103	0	
	2/3	17	30	p<0.0001(b)
Stage	I/II	53	56	
	III/IV	1	10	p = 0.028(b)
LVSI	Absent	99	13	
	Present	21	17	p<0.0001(b)
Recurrence/Metastasis	Absent	110	17	
	Present	10	13	p<0.0001(b)
Nuclear YAP score	0	23	1	
	1	31	2	
	2	20	8	
	3	16	7	
	4	30	12	
	Low (score: 0/1)	54	3	
	High (score: 2/3/4)	66	27	p = 0.0009(b)
Cytoplasmic YAP score	0	3	0	
	1	14	0	
	2	21	11	
	3	40	9	
	4	42	10	
	Low (score: 0/1/2)	38	11	
	High (score: 3/4)	82	19	N.S.(b)

BMI; Body mass index, LVSI; Lymphovascular space involvement, N.S.; not significant.

(a): Student's t-test, (b): Chi square test (there is no <10 number in data)/Fisher's exact test (there is <10 number in data).

**Table 2:** Association of YAP expression in nucleus with clinicopathological factors in type1.

		Type1 cancer (n = 120)		
		Nuclear YAP expression		
		Low (Score; 0/1) (n = 54)	High (Score; 2/3/4) (n = 66)	P-value
Age (year)	mean (range)	62.8 (40–89)	64.2 (44–82)	N.S.(a)
BMI	mean (range)	34.8 (19.4–54.5)	38.4 (19.1–70.4)	N.S.(a)
Ethnic group	White	54	65	
	Other races	0	1	N.S.(b)
Grade	1	39	29	
	2/3	15	37	p = 0.019(b)
Stage	I/II	53	56	
	III/IV	1	10	p = 0.028(b)
LVSI	Absent	52	47	
	Present	2	19	p = 0.0008(b)
Recurrence/Metastasis	Absent	53	57	
	Present	1	9	p = 0.046(b)

BMI; Body mass index, LVSI; Lymphovascular space involvement, N.S.; not significant.

(a): Student's t-test, (b): Chi square test (there is no <10 number in data)/Fisher's exact test (there is <10 number in data).

### YAP protein expression in EMCA cell lines

**Figure 2:**
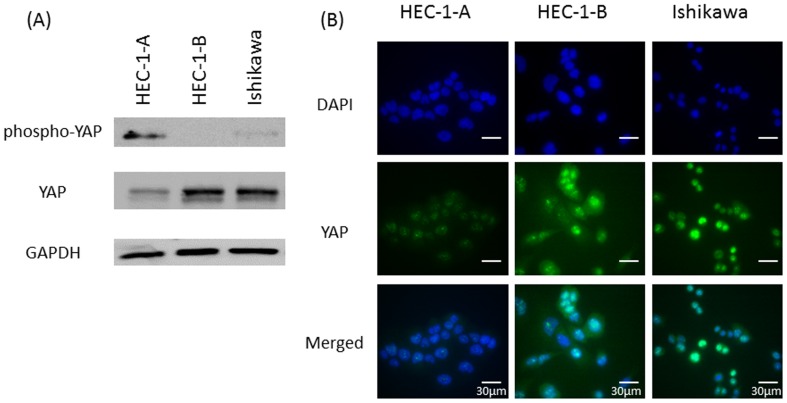
(A) Expression of YAP and phospho-YAP (Ser127) in three EMCA cell lines by western blotting. (B) Immunofluorescent cytochemical staining of endogenous YAP using anti-YAP antibody (YAP: green, DAPI: blue).

To gain further insight into the biological roles of YAP in EMCA, we chose to use three type 1 EMCA cell lines; HEC-1-A, HEC-1-B and Ishikawa. We first evaluated protein levels of YAP and phospho-YAP (inactive YAP) (Figure 2A). HEC-1-B demonstrated the highest levels of total YAP and very low levels of inactive phospho-YAP by western blotting. In contrast, HEC-1-A expressed the lowest levels of total YAP and the highest levels of inactive phospho-YAP. Immunofluorescence confirmed high levels of nuclear and cytoplasmic YAP in HEC-1-B cells and low levels of nuclear and cytoplasmic YAP in HEC-1-A cells, consistent with the results of western blotting (Figure 2B). Therefore, HEC-1-B cells were used for a loss-of-function experiment, and conversely HEC-1-A cells were used for a gain-of-function experiment.

### YAP knockdown in EMCA cells

**Figure 3:**
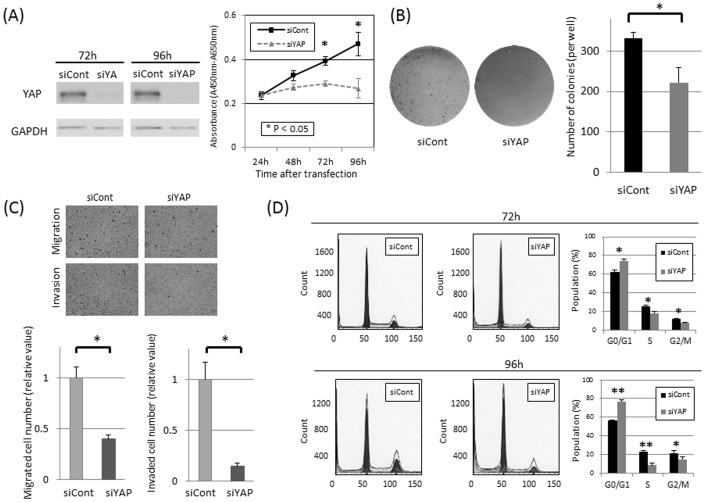
(A) Inhibited protein expression of YAP by YAP-specific siRNA (siYAP) was confirmed by western blotting both 72 and 96 hours after transfection (left). The number of viable cells between 24–96 hours after transfection of siYAP or control siRNA (siCont) was evaluated in HEC-1-B cells using the water-soluble tetrazolium salt assay (Cell counting kit-8) (right). 26.2% and 42.9% inhibition of cell proliferation were confirmed by knockdown of YAP expression at 72 and 96 hours, respectively. (* P<0.05 (the Mann-Whitney u-test)). Results are shown in means ±standard deviations (bars) in quadruplicate experiments. Similar trends were obtained in other two independent experiments. (B) The number of colonies in soft agar was compared between the YAP inhibited cells and the control cells to evaluate anchorage-independent cell growth in HEC-1-B cells. Representative image of formed colonies in soft agar (left). Quantitative analysis in number of formed colonies (right), showing a significant decrease in siYAP transfected cells. (* P<0.05 (the Mann-Whitney u-test)). Columns and bars represent means and standard deviation in sextuplicate experiments. Similar trends were obtained in another independent experiment. (C) Transwell migration and invasion assay. Representative image of migrated/invaded HEC-1-B cells (left). Quantitative analysis in number of migrated/invaded cells (right), which showed a significant decrease in siYAP transfected cells. (* P<0.05 (the Mann-Whitney u-test)). Columns and bars represent means and standard deviation in quadruplicate experiments. Similar trends were obtained in another independent experiment. (D) Flow cytometric analysis. Representative results of the population in each phase of the cell cycle in HEC-1-B EMCA cells 72 and 96 hours after transfection with siCont/siYAP (left). Quantitative analysis in the population in each phase (right). siYAP transfected cells showed a significant accumulation of cells in G0/G1 phase and significant decreases in S and G2/M phases at 72 and 96 hours (* P<0.05, ** P<0.001, the Student's t- test). Columns and bars represent means and standard deviation in triplicate experiments. Similar trends were obtained in another independent experiment.

To extend our immunohistochemical results demonstrating a correlation between nuclear YAP and poor prognostic features in EMCA patients, we determined the effects of YAP knockdown on cell proliferation using YAP-specific siRNA (siYAP) and control siRNA (siCont) in HEC-1-B EMCA cells. Endogenous expression of the YAP protein was efficiently inhibited at 72 and 96 hours after transient transfection of siRNA (Figure 3A). Cell proliferation was significantly decreased in the YAP inhibited cells compared with the control cells by 26.2% (72 hours) and 42.9% (96 hours), respectively (Figure 3A). This similar growth inhibitory effect in cell proliferation was confirmed using an alternate YAP siRNA sequence (data not shown).

We also determined the role of YAP in anchorage-independent cell growth, migration and invasion ability, commonly considered characteristics of malignant transformed cells. In soft agar colony formation assays, knockdown of YAP in HEC-1-B cells decreased the ability of colony formation compared to control cells (p = 0.015, Figure 3B). In cell migration/invasion assays, a significant decrease in the number of cells that migrated/invaded through uncoated/Matrigel-coated membranes was observed in siYAP transfected cells compared with siCont transfected cells (p<0.05, Figure 3C). These experiments demonstrate that YAP expression is associated with cell proliferation, anchorage-independent growth and migration/invasion potential in this EMCA cell line.

### Cell cycle analysis in EMCA cells

To determine whether growth inhibition induced by YAP knockdown caused alterations in the cell cycle, flow cytometric analysis was performed in HEC-1-B EMCA cells transfected with YAP-specific and control siRNAs. Consistent with the results of the cell proliferation assays, siYAP treated HEC-1-B cells exhibited a significant accumulation of cells in G0/G1 phase and significant decreases in S and G2/M phases at 72 and 96 hours, compared with siCont treated cells (Figure 3D). Taken together, these data suggest that YAP knockdown produces G0-G1 cell cycle arrest in this EMCA cell line.

### YAP overexpression in EMCA cells

**Figure 4:**
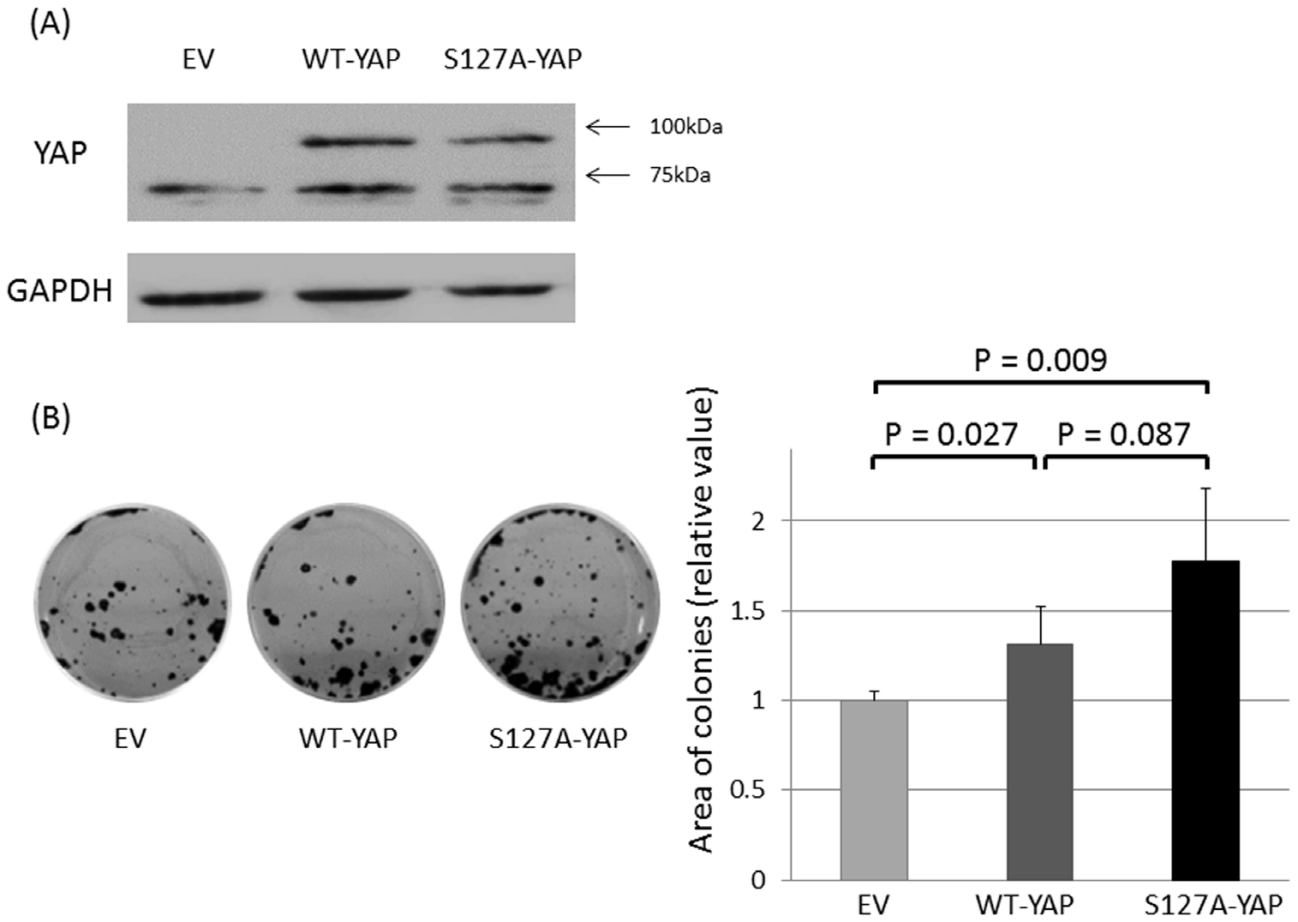
(A) Endogenous expression of YAP (approximately 65 kDa) and exogenous expression of YAP tagged with GFP protein (approximately 92 kDa in total) in HEC-1-A cells transfected with wild-type YAP (WT-YAP) and S127A mutant YAP (S127A-YAP). (B) Representative image of colony formation assay (left). Cells were transiently transfected with empty vector (EV)/WT-YAP/S127A-YAP and selected with appropriate concentrations of G418 for four weeks. The drug-resistant colonies formed by the YAP-transfected cells were significantly numerous compared with the control (the Mann-Whitney U-test). Columns and bars represent means and standard deviation in quadruplicate experiments. Similar trends were obtained in another independent experiment.

Based on our results from the loss-of-function assays, we hypothesized that overexpression of YAP in EMCA cells with low endogenous YAP levels would increase cell proliferation. We investigated the ability of forming colonies after transient transfection of YAP expressing constructs in HEC-1-A cells, which exhibited relatively low levels of YAP and high levels of phospho-YAP (Figure 2). Overexpression of GFP-tagged wild-type YAP and GFP-tagged constitutively active S127A mutant YAP in HEC-1-A cells was verified by western blotting (Figure 4A). Cells overexpressing WT-YAP or S127A-YAP produced significantly more colonies compared with cells transfected with the control empty vector (EV). While S127A-YAP transfected cells produced the most colonies, a significant difference was not observed compared to WT-YAP transfected cells (Figure 4B). These results further support a role for YAP in cell proliferation of EMCA.

### Role of YAP in modulating radiation sensitivity

**Figure 5:**
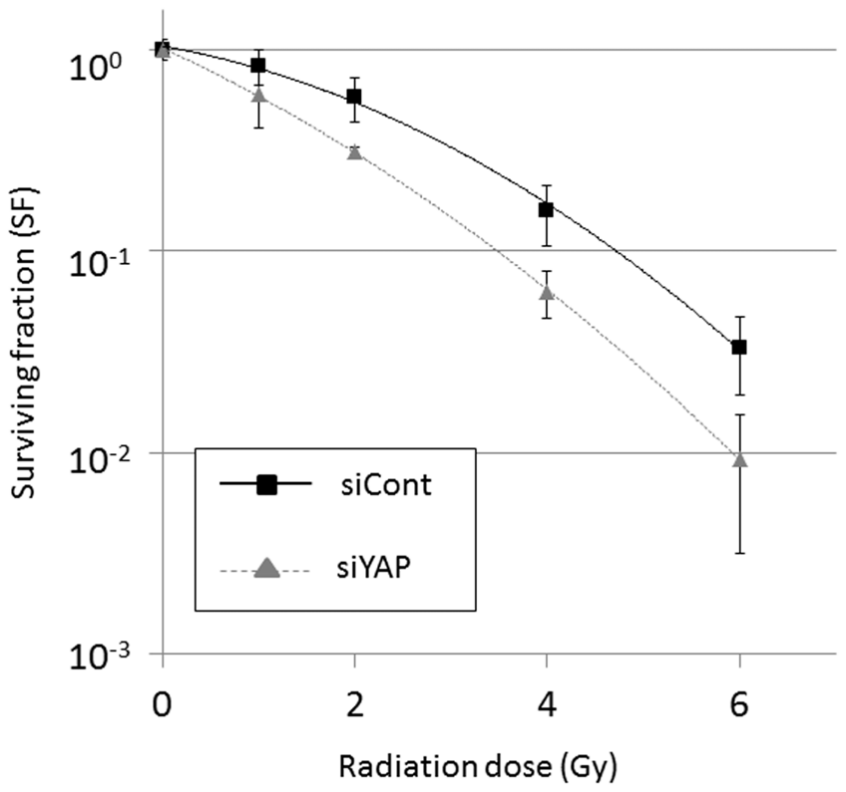
Clonogenic assay in HEC-1-B cells after radiation exposure. Knockdown of YAP expression by siRNA reduced clonogenic survival in HEC-1-B cells, resulting in an increase in radiation sensitivity with a dose enhancement factor at 10% survival (DEF 0.1) of 1.36. Results are shown in means ±standard deviations (bars) in triplicate experiments. Similar trends were obtained in other three independent experiments.

Given the importance of radiation therapy in EMCA treatment and recently published data implicating YAP in modulating the radiation sensitivity of medulloblastoma cells, we next determined the effect of YAP on radiation sensitivity in EMCA cells using a clonogenic survival assay. Knockdown of YAP expression by siRNA reduced clonogenic survival in HEC-1-B cells, resulting in an increase in radiation sensitivity with a DEF 0.1 of 1.36 (**Figure 5**). Specifically, 90% cell killing by radiation exposure in siYAP transfected cells required 3.48 Gy, whereas the same level of cell killing in siCont transfected cells required 4.72 Gy. Therefore, our results demonstrate an increased sensitivity to radiation with loss of YAP expression in HEC-1-B EMCA cells.

## Discussion

Our data identify the oncogenic functions of YAP in EMCA. The Hippo pathway, especially the direct upstream regulator, LATS1/2, negatively modulates YAP activity by phosphorylation of its S127 site. Phosphorylated-YAP is segregated in the cytoplasm and targeted for ubiquitin-mediated proteolysis [43], [44]. Therefore, the subcellular localization of YAP has been considered crucial in determining its biological functions in various cancers [42]. Nuclear YAP expression as a poor prognostic marker has been shown in other types of cancer, such as esophageal, gastric and ovarian [22], [28], [31], [32]. Our immunohistochemical studies showed that higher levels of nuclear YAP were associated with poor prognostic factors, such as advanced stage, grade, LVSI, postoperative recurrence/metastasis and overall survival. Our findings that YAP nuclear staining is an independent prognostic factor in overall survival is unique for a disease that at an early stage has a 80–95% 5-year survival rate. Interestingly, cytoplasmic expression of YAP was not significantly associated with similar histologic features or prognosis, indicating that subcellular localization of YAP is important not only in biological functions as an oncogene, but also as a prognostic indicator in primary EMCA tissues. Larger scale studies involving cooperative group trials such as the Gynecologic Oncology Group may be necessary to confirm and extend our findings.

YAP can be either proliferative or apoptotic in different cell contexts. Our data demonstrate that YAP expression is correlated with cell proliferation, anchorage independent growth, invasion and migration in the context of EMCA. Inhibition of cell proliferation in response to knockdown of YAP expression is associated with an increase of cells in G0/G1 phase of the cell cycle and a decrease of cells in S phase. Consistent with our results, previous reports have demonstrated that altered expression of the Hippo pathway, including YAP, alters cell cycle check point mechanisms [22], [45], [46].

The mechanism of YAP activation in EMCA is currently unknown. The *YAP* gene is located at chromosome 11q22 and several reports have identified amplification of this locus in breast, esophageal, hepatocellular cancer, ependymoma, malignant mesothelioma and medulloblastoma [21], [22], [23], [24], [25], [26]. Amplification of 11q22 has not been identified in EMCA, based on previous comprehensive analyses of somatic alterations [47], [48]. Other factors independent of the increased gene copy number, such as the modulation of gene transcription, protein translation, or RNA/protein stability likely contribute to enhanced protein expression of YAP in EMCA.

We are currently exploring the possibility of YAP activating mutations and alterations in upstream regulators of the Hippo pathway in primary human EMCA and in cultured cell lines. Understanding the mechanism of YAP regulation and its downstream targets will be important in therapeutic targeting of YAP action. Dhananjayan et al. have demonstrated the role of YAP as a co-activator of the ER and PR through cooperation with WBP-2, suggesting that YAP can modulate the transcriptional activity of steroid hormone receptors [33]. Thus, we hypothesize that YAP may act in coordination with ER/PR to regulate cell specific target genes in EMCA. The oncogenic properties in EMCA may be explained by the complexity and multiple functions of YAP regulated genes.

The contribution of YAP to radiosensitivity has been reported previously only in medulloblastoma by Fernandez-L et al., who showed enhanced proliferation in YAP overexpressing cells after radiation exposure [34]. This effect was driven by activation of insulin-like growth factor 2 (IGF2) and Akt, as well as subsequent modulations of G1/S and G2/M checkpoints after irradiation. In our study, YAP knockdown in the HEC-1-B EMCA cell line promoted radiosensitivity compared with the control group, consistent with the findings of Fernandez-L et al [34]. Furthermore, those findings are also consistent with our flow cytometric analysis, which showed a decrease of S phase cells after YAP knockdown; the S phase is generally recognized as the most radioresistant segment of the cell cycle [49], [50]. While the radiation doses in our assays do not mimic doses or schedules used in human subjects, our data suggest that higher levels of YAP promote radiation resistance. Xenograft mouse models are planned for further exploring the role of YAP in modulating radiation sensitivity. Fernandez et al. identified altered phosphorylation of DNA damage response (DDR) proteins, such as ATM and Chk2, after radiation exposure in medulloblastoma. We were unable to demonstrate reproducible alterations in expression of DDR proteins, such as ATM, ATR, Chk1, Chk2 and p53 in our model system (data not shown). Further elucidation of the mechanism of YAP and radiosensitivity will require global genome based evaluation.

Our findings shed light on a novel biological and oncogenic function of YAP in EMCA, and suggest that development of Hippo pathway-based therapeutic strategies to overcome radiation resistance and inhibit cell proliferation would be useful in EMCA therapy. In conclusion, this study is the first to demonstrate biological and clinical features of YAP as an oncogenic protein in EMCA. Further investigation will clarify its role as a therapeutic target, a diagnostic marker and an indicator of appropriate therapy for an individual EMCA patient.

## Supporting Information

Figure S1 **Representative micrographs in immunohistochemical staining of YAP.** (A) Type 1 cancer with grade 1: moderate cytoplasmic staining (+1 to +2), but no nuclear stain (0). (B) Type 1 cancer with grade 2: variable nuclear and cytoplasmic staining levels, ranging between +2 and +4. (C) Type 1 cancer with grade 3: diffuse and strong staining levels of YAP in both nucleus (4+) and cytoplasm (4+). (D) Type 2 cancer, carcinosarcoma: strong staining levels of YAP in both nucleus (4+) and cytoplasm (+4).
(TIF)

Figure S2 **The Kaplan-Meier curves for overall survival rate of patients with type 1 EMCA patients (n = 120) according to stage (left) and LVSI (right).** Advanced stage (III/IV) and presence of LVSI were significantly associated with a worse survival (P = 0.0005 and 0.003, respectively, log-rank test).
(TIF)

Figure S3 (A) The Kaplan-Meier curve for overall survival rate of patients with type 1 EMCA (n = 120) according to the nuclear expression of YAP. (B, C) The Kaplan-Meier curves for overall survival rate of patients with type 2 (n = 30) according to the nuclear/cytoplasmic expression of YAP. No significant correlations were observed regarding overall survival in cytoplasmic YAP in type 1 cancer and nuclear/cytoplasmic YAP in type 2 cancer.
(TIF)

Table S1 **Association of YAP expression in nucleus with clinicopathological factors in type2 endometrial cancers.**
(TIF)

## References

[1] Siegel R, Naishadham D, Jemal A (2013) Cancer statistics, 2013. CA Cancer J Clin 63: 11–30. 23335087 10.3322/caac.21166

[2] Howlader N, Noone A, Krapcho M, Garshell J, Neyman N, et al. (2013) SEER Cancer Statistics Review, 1975–2010. Bethesda, MD: National Cancer Institute, http://seer.cancer.gov/csr/1975_2010/, [Based on the November 2012 SEER data submission, posted to the SEER web site, April 2013. http://seer.cancer.gov/statfacts/html/corp.html].

[3] Fader AN, Arriba LN, Frasure HE, von Gruenigen VE (2009) Endometrial cancer and obesity: epidemiology, biomarkers, prevention and survivorship. Gynecol Oncol 114: 121–127. 19406460 10.1016/j.ygyno.2009.03.039

[4] Byron SA, Gartside M, Powell MA, Wellens CL, Gao F, et al (2012) FGFR2 point mutations in 466 endometrioid endometrial tumors: relationship with MSI, KRAS, PIK3CA, CTNNB1 mutations and clinicopathological features. PLoS One 7: e30801. 22383975 10.1371/journal.pone.0030801 PMC3285611

[5] Tashiro H, Blazes MS, Wu R, Cho KR, Bose S, et al (1997) Mutations in PTEN are frequent in endometrial carcinoma but rare in other common gynecological malignancies. Cancer Res 57: 3935–3940. 9307275

[6] Rudd ML, Price JC, Fogoros S, Godwin AK, Sgroi DC, et al (2011) A unique spectrum of somatic PIK3CA (p110alpha) mutations within primary endometrial carcinomas. Clin Cancer Res 17: 1331–1340. 21266528 10.1158/1078-0432.CCR-10-0540 PMC3060282

[7] Dutt A, Salvesen HB, Chen TH, Ramos AH, Onofrio RC, et al (2008) Drug-sensitive FGFR2 mutations in endometrial carcinoma. Proc Natl Acad Sci U S A 105: 8713–8717. 18552176 10.1073/pnas.0803379105 PMC2438391

[8] Clement PB, Young RH (2002) Endometrioid carcinoma of the uterine corpus: a review of its pathology with emphasis on recent advances and problematic aspects. Adv Anat Pathol 9: 145–184. 11981113 10.1097/00125480-200205000-00001

[9] Clement PB, Young RH (2004) Non-endometrioid carcinomas of the uterine corpus: a review of their pathology with emphasis on recent advances and problematic aspects. Adv Anat Pathol 11: 117–142. 15096727 10.1097/00125480-200405000-00001

[10] Morrow CP, Bundy BN, Kurman RJ, Creasman WT, Heller P, et al (1991) Relationship between surgical-pathological risk factors and outcome in clinical stage I and II carcinoma of the endometrium: a Gynecologic Oncology Group study. Gynecol Oncol 40: 55–65. 1989916 10.1016/0090-8258(91)90086-k

[11] Creutzberg CL, van Putten WL, Koper PC, Lybeert ML, Jobsen JJ, et al (2000) Surgery and postoperative radiotherapy versus surgery alone for patients with stage-1 endometrial carcinoma: multicentre randomised trial. PORTEC Study Group. Post Operative Radiation Therapy in Endometrial Carcinoma. Lancet 355: 1404–1411. 10791524 10.1016/s0140-6736(00)02139-5

[12] Keys HM, Roberts JA, Brunetto VL, Zaino RJ, Spirtos NM, et al (2004) A phase III trial of surgery with or without adjunctive external pelvic radiation therapy in intermediate risk endometrial adenocarcinoma: a Gynecologic Oncology Group study. Gynecol Oncol 92: 744–751. 14984936 10.1016/j.ygyno.2003.11.048

[13] Creutzberg CL, van Putten WL, Koper PC, Lybeert ML, Jobsen JJ, et al (2003) Survival after relapse in patients with endometrial cancer: results from a randomized trial. Gynecol Oncol 89: 201–209. 12713981 10.1016/s0090-8258(03)00126-4

[14] Sudol M (1994) Yes-associated protein (YAP65) is a proline-rich phosphoprotein that binds to the SH3 domain of the Yes proto-oncogene product. Oncogene 9: 2145–2152. 8035999

[15] Sudol M, Gelman IH, Zhang J (2013) YAP1 Uses Its Modular Protein Domains and Conserved Sequence Motifs to Orchestrate Diverse Repertoires of Signaling. The Hippo Signaling Pathway and Cancer: Springer. pp. 53–70.

[16] Zhao B, Ye X, Yu J, Li L, Li W, et al (2008) TEAD mediates YAP-dependent gene induction and growth control. Genes Dev 22: 1962–1971. 18579750 10.1101/gad.1664408 PMC2492741

[17] Zhang J, Ji JY, Yu M, Overholtzer M, Smolen GA, et al (2009) YAP-dependent induction of amphiregulin identifies a non-cell-autonomous component of the Hippo pathway. Nat Cell Biol 11: 1444–1450. 19935651 10.1038/ncb1993 PMC2819909

[18] Wang H, Du YC, Zhou XJ, Liu H, Tang SC (2013) The dual functions of YAP-1 to promote and inhibit cell growth in human malignancy. Cancer Metastasis Rev. 10.1007/s10555-013-9463-3 24346160

[19] Harvey KF, Zhang X, Thomas DM (2013) The Hippo pathway and human cancer. Nat Rev Cancer 13: 246–257. 23467301 10.1038/nrc3458

[20] Oka T, Mazack V, Sudol M (2008) Mst2 and Lats kinases regulate apoptotic function of Yes kinase-associated protein (YAP). J Biol Chem 283: 27534–27546. 18640976 10.1074/jbc.M804380200

[21] Overholtzer M, Zhang J, Smolen GA, Muir B, Li W, et al (2006) Transforming properties of YAP, a candidate oncogene on the chromosome 11q22 amplicon. Proc Natl Acad Sci U S A 103: 12405–12410. 16894141 10.1073/pnas.0605579103 PMC1533802

[22] Muramatsu T, Imoto I, Matsui T, Kozaki K, Haruki S, et al (2011) YAP is a candidate oncogene for esophageal squamous cell carcinoma. Carcinogenesis 32: 389–398. 21112960 10.1093/carcin/bgq254

[23] Zender L, Spector MS, Xue W, Flemming P, Cordon-Cardo C, et al (2006) Identification and validation of oncogenes in liver cancer using an integrative oncogenomic approach. Cell 125: 1253–1267. 16814713 10.1016/j.cell.2006.05.030 PMC3026384

[24] Modena P, Lualdi E, Facchinetti F, Veltman J, Reid JF, et al (2006) Identification of tumor-specific molecular signatures in intracranial ependymoma and association with clinical characteristics. J Clin Oncol 24: 5223–5233. 17114655 10.1200/JCO.2006.06.3701

[25] Yokoyama T, Osada H, Murakami H, Tatematsu Y, Taniguchi T, et al (2008) YAP1 is involved in mesothelioma development and negatively regulated by Merlin through phosphorylation. Carcinogenesis 29: 2139–2146. 18725387 10.1093/carcin/bgn200

[26] Fernandez LA, Northcott PA, Dalton J, Fraga C, Ellison D, et al (2009) YAP1 is amplified and up-regulated in hedgehog-associated medulloblastomas and mediates Sonic hedgehog-driven neural precursor proliferation. Genes Dev 23: 2729–2741. 19952108 10.1101/gad.1824509 PMC2788333

[27] Wang L, Shi S, Guo Z, Zhang X, Han S, et al (2013) Overexpression of YAP and TAZ is an independent predictor of prognosis in colorectal cancer and related to the proliferation and metastasis of colon cancer cells. PLoS One 8: e65539. 23762387 10.1371/journal.pone.0065539 PMC3677905

[28] Kang W, Tong JH, Chan AW, Lee TL, Lung RW, et al (2011) Yes-associated protein 1 exhibits oncogenic property in gastric cancer and its nuclear accumulation associates with poor prognosis. Clin Cancer Res 17: 2130–2139. 21346147 10.1158/1078-0432.CCR-10-2467

[29] Xu MZ, Yao TJ, Lee NP, Ng IO, Chan YT, et al (2009) Yes-associated protein is an independent prognostic marker in hepatocellular carcinoma. Cancer 115: 4576–4585. 19551889 10.1002/cncr.24495 PMC2811690

[30] Wang Y, Dong Q, Zhang Q, Li Z, Wang E, et al (2010) Overexpression of yes-associated protein contributes to progression and poor prognosis of non-small-cell lung cancer. Cancer Sci 101: 1279–1285. 20219076 10.1111/j.1349-7006.2010.01511.x PMC11158334

[31] Hall CA, Wang R, Miao J, Oliva E, Shen X, et al (2010) Hippo pathway effector Yap is an ovarian cancer oncogene. Cancer Res 70: 8517–8525. 20947521 10.1158/0008-5472.CAN-10-1242 PMC2970655

[32] Zhang X, George J, Deb S, Degoutin JL, Takano EA, et al (2011) The Hippo pathway transcriptional co-activator, YAP, is an ovarian cancer oncogene. Oncogene 30: 2810–2822. 21317925 10.1038/onc.2011.8

[33] Dhananjayan SC, Ramamoorthy S, Khan OY, Ismail A, Sun J, et al (2006) WW domain binding protein-2, an E6-associated protein interacting protein, acts as a coactivator of estrogen and progesterone receptors. Mol Endocrinol 20: 2343–2354. 16772533 10.1210/me.2005-0533

[34] Fernandez LA, Squatrito M, Northcott P, Awan A, Holland EC, et al (2012) Oncogenic YAP promotes radioresistance and genomic instability in medulloblastoma through IGF2-mediated Akt activation. Oncogene 31: 1923–1937. 21874045 10.1038/onc.2011.379 PMC3583298

[35] Creasman W (1989) Announcement, FIGO stages 1988, revisions. Gynecol Oncol 35: 7.

[36] Mutch DG (2009) The new FIGO staging system for cancers of the vulva, cervix, endometrium and sarcomas. Gynecologic oncology 115: 325–328.

[37] Singh M, Spoelstra NS, Jean A, Howe E, Torkko KC, et al (2008) ZEB1 expression in type I vs type II endometrial cancers: a marker of aggressive disease. Mod Pathol 21: 912–923. 18487993 10.1038/modpathol.2008.82

[38] Cochrane DR, Spoelstra NS, Howe EN, Nordeen SK, Richer JK (2009) MicroRNA-200c mitigates invasiveness and restores sensitivity to microtubule-targeting chemotherapeutic agents. Mol Cancer Ther 8: 1055–1066. 19435871 10.1158/1535-7163.MCT-08-1046 PMC4573391

[39] Albitar L, Pickett G, Morgan M, Davies S, Leslie KK (2007) Models representing type I and type II human endometrial cancers: Ishikawa H and Hec50co cells. Gynecol Oncol 106: 52–64. 17490735 10.1016/j.ygyno.2007.02.033

[40] Nishimura Y, Komatsu S, Ichikawa D, Nagata H, Hirajima S, et al (2013) Overexpression of YWHAZ relates to tumor cell proliferation and malignant outcome of gastric carcinoma. Br J Cancer 108: 1324–1331. 23422756 10.1038/bjc.2013.65 PMC3619260

[41] Sudol M (2013) YAP1 oncogene and its eight isoforms. Oncogene 32: 3922. 23160371 10.1038/onc.2012.520

[42] Steinhardt AA, Gayyed MF, Klein AP, Dong J, Maitra A, et al (2008) Expression of Yes-associated protein in common solid tumors. Hum Pathol 39: 1582–1589. 18703216 10.1016/j.humpath.2008.04.012 PMC2720436

[43] Zhao B, Wei X, Li W, Udan RS, Yang Q, et al (2007) Inactivation of YAP oncoprotein by the Hippo pathway is involved in cell contact inhibition and tissue growth control. Genes Dev 21: 2747–2761. 17974916 10.1101/gad.1602907 PMC2045129

[44] Dong J, Feldmann G, Huang J, Wu S, Zhang N, et al (2007) Elucidation of a universal size-control mechanism in Drosophila and mammals. Cell 130: 1120–1133. 17889654 10.1016/j.cell.2007.07.019 PMC2666353

[45] Aylon Y, Michael D, Shmueli A, Yabuta N, Nojima H, et al (2006) A positive feedback loop between the p53 and Lats2 tumor suppressors prevents tetraploidization. Genes Dev 20: 2687–2700. 17015431 10.1101/gad.1447006 PMC1578695

[46] Tschop K, Conery AR, Litovchick L, Decaprio JA, Settleman J, et al (2011) A kinase shRNA screen links LATS2 and the pRB tumor suppressor. Genes Dev 25: 814–830. 21498571 10.1101/gad.2000211 PMC3078707

[47] Salvesen HB, Carter SL, Mannelqvist M, Dutt A, Getz G, et al (2009) Integrated genomic profiling of endometrial carcinoma associates aggressive tumors with indicators of PI3 kinase activation. Proc Natl Acad Sci U S A 106: 4834–4839. 19261849 10.1073/pnas.0806514106 PMC2660768

[48] Kandoth C, Schultz N, Cherniack AD, Akbani R, Liu Y, et al (2013) Integrated genomic characterization of endometrial carcinoma. Nature 497: 67–73. 23636398 10.1038/nature12113 PMC3704730

[49] Pawlik TM, Keyomarsi K (2004) Role of cell cycle in mediating sensitivity to radiotherapy. Int J Radiat Oncol Biol Phys 59: 928–942. 15234026 10.1016/j.ijrobp.2004.03.005

[50] Pajonk F, Vlashi E, McBride WH (2010) Radiation resistance of cancer stem cells: the 4 R's of radiobiology revisited. Stem Cells 28: 639–648. 20135685 10.1002/stem.318 PMC2940232

