# Supplementary figures and images for: Yes-Associated Protein (YAP) Modulates Oncogenic Features and Radiation Sensitivity in Endometrial Cancer
Source: PLoS One. 2014 Jun 27;9(6):e100974. doi: 10.1371/journal.pone.0100974 (PMC4074125; doi:10.1371/journal.pone.0100974)

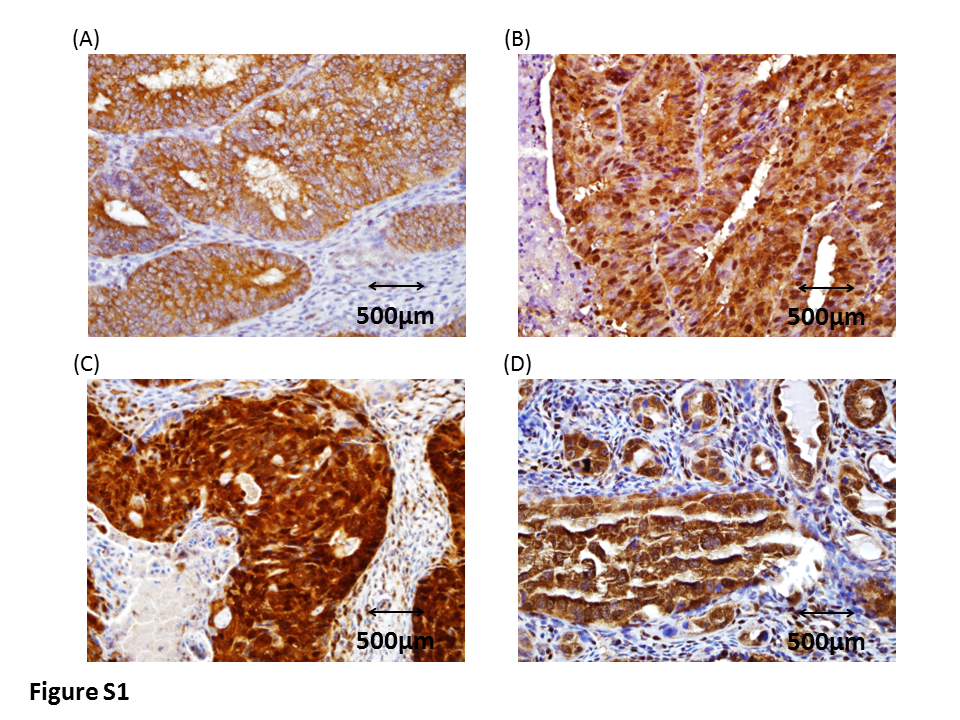

Supplement: Figure S1 — Representative micrographs in immunohistochemical staining of YAP. (A) Type 1 cancer with grade 1: moderate cytoplasmic staining (+1 to +2), but no nuclear stain (0). (B) Type 1 cancer with grade 2: variable nuclear and cytoplasmic staining levels, ranging between +2 and +4. (C) Type 1 cancer with grade 3: diffuse and strong staining levels of YAP in both nucleus (4+) and cytoplasm (4+). (D) Type 2 cancer, carcinosarcoma: strong staining levels of YAP in both nucleus (4+) and cytoplasm (+4). (TIF) [file pone.0100974.s001.tif]
